# Characterization and Monitoring of Titanium Bone Implants with Impedance Spectroscopy

**DOI:** 10.3390/s20164358

**Published:** 2020-08-05

**Authors:** Alberto Olmo, Miguel Hernández, Ernesto Chicardi, Yadir Torres

**Affiliations:** 1 Instituto de Microelectrónica de Sevilla, IMSE-CNM (CSIC, Universidad de Sevilla), Av. Américo Vespucio, sn, 41092 Sevilla, Spain; miguelhernandezcamacho@gmail.com; 2 Escuela Técnica Superior de Ingeniería Informática, Departamento de Tecnología Electrónica, Universidad de Sevilla, Av. Reina Mercedes sn, 41012 Sevilla, Spain; 3 Departamento de Ingeniería y Ciencia de los Materiales y del Transporte, Escuela Superior de Ingenieros, Universidad de Sevilla, 41092 Sevilla, Spain; echicardi@us.es; 4 Departamento de Ingeniería y Ciencia de los Materiales y del Transporte, Escuela Politécnica Superior, Calle Virgen de África, 7, 41011 Sevilla, Spain; ytorres@us.es

**Keywords:** electrical impedance spectroscopy, smart implants, cortical bone tissue, porous titanium

## Abstract

Porous titanium is a metallic biomaterial with good properties for the clinical repair of cortical bone tissue, although the presence of pores can compromise its mechanical behavior and clinical use. It is therefore necessary to characterize the implant pore size and distribution in a suitable way. In this work, we explore the new use of electrical impedance spectroscopy for the characterization and monitoring of titanium bone implants. Electrical impedance spectroscopy has been used as a non-invasive route to characterize the volumetric porosity percentage (30%, 40%, 50% and 60%) and the range of pore size (100–200 and 355–500 mm) of porous titanium samples obtained with the space-holder technique. Impedance spectroscopy is proved to be an appropriate technique to characterize the level of porosity of the titanium samples and pore size, in an affordable and non-invasive way. The technique could also be used in smart implants to detect changes in the service life of the material, such as the appearance of fractures, the adhesion of osteoblasts and bacteria, or the formation of bone tissue.

## 1. Introduction

Electrical impedance measurements have long been used in biomedical engineering [1], and they are also receiving recent interest for a wide variety of applications, such as cardiovascular diagnosis [2], prostheses osseointegration assessment [3], wearable medical devices [4], or 3D image analysis for the detection of medical anomalies, such as cancer [5].

Clinical repair of bone tissue is a worldwide healthcare challenge, due to the rising incidence of ageing and related diseases, cancer, or traffic accidents, among other reasons [6]. In many cases, the correction of bone defects is difficult, and an orthopedic prosthesis is necessary to enable the functional recovery and its biocompatibility with the host tissue.

Titanium has been commonly used in orthopedic prostheses due to its unique biomedical properties, such as high specific resistance, low weight, and its high corrosion resistance, but pure titanium is a material unable to properly emulate the mechanical requirements of the bone [7]. For this reason, porous materials have been developed to correct those problems that occurred in solid materials, offering better similarities in their values of Young’s modulus, closer to that of the human bone. The main techniques currently used for the creation of porous structures in biomaterials are the partial consolidation (loose sintering), the space-holders technique or the injection of gases in molten metals [7]. Among them, the space-holders technique presents promising results compared to other techniques based on powder metallurgy [8], but it is, however, necessary to determine the correct pore distribution and mechanical features for the fabricated porous structure before its implantation and monitor its evolution.

The two main methods currently used to determine the porosity for compacted or sintered specimens by powder metallurgy are the Archimedes method and image analysis using an image processing software. The main drawback with the Archimedes method is the determination of only a global porosity value. In contrast, for image analysis, the destruction of the piece is previously required [9], being then an undesirable destructive analysis. None of these techniques provide a practical method for the real-time monitoring of porous titanium bone implants. In addition, in many cases these techniques lack of statistical significance to effectively discriminate the volumetric porosity or the size range, for similar specimens [8,9].

In this work, we explored the use of electrical impedance spectroscopy to characterize the porosity of titanium bone implants and their possible application in the monitoring of the implantation. Initial experiments were developed in [10] to correlate porosity of titanium, size of pore, and electrical conductivity, and an empirical model was developed. However, this work does not describe the influence of the electric frequency in the measured impedance, as it would be required for the use of electrical impedance spectroscopy. The oxide layer that is formed on the titanium surface in in vivo conditions has also been studied in [11,12], and an electrical model has been proposed to characterize the corrosion processes occurring in in vivo situations. The influence of pore morphology on the corrosion of porous titanium implants was previously studied in [13]. In this study, small and isolated pores seemed to encourage the stagnation of electrolytes, preventing their free flow crucial to the ion incorporation/titanium release process of passivation. In [14], on another hand, it is shown through electrochemical impedance spectroscopy that the pores in the porous titanium play a negative part in corrosion resistance, and the flowing electrolyte can increase the corrosive rate of all titanium samples. It is therefore necessary to conduct more experiments to evaluate the influence of pores on the titanium electrochemical properties.

We present in this work the use of electrical impedance as a method to characterize the porosity of the structure, in a non-invasive and affordable way, with the potential to create 3D maps of the implant (known as electrical impedance spectroscopy [5]). For this objective, it was first needed to study the sensitivity of electrical impedance spectroscopy to changes in the porosity of titanium samples.

An initial characterization work in different titanium samples is presented, with different sizes of pores and different degrees of porosity in volume. Electrical impedance measurements are compared with other techniques to compare the porosity at different levels (superficial and volumetric). A detailed analysis of impedance spectroscopy results is presented, studying the precision of the method to discriminate between different specimens. An electrical model is finally presented, to correctly understand the experimental values obtained.

Furthermore, electrical impedance spectroscopy has also been proposed to characterize the quality of the tissue at the interface between the bone and the prosthesis [3], or for tissue classification [15]. The use of electrical impedance spectroscopy for cell culture monitoring is also studied in our work, analyzing different electrical models used and analyzing a combined strategy for measuring both the state of the implant and its osseointegration, which would be an important advance on the state-of-the-art model.

The design of a low-cost microcontroller-based instrument for prostheses osseointegration assessment has also been proposed in [16], based on impedance spectroscopy. A similar low-cost design that could also assess the state of the implant porous structure can be proposed, for a more complete assessment of the implant, as a medical sensor.

## 2. Materials and Methods

### 2.1. Porous Samples Preparation

The chemical composition of metallic initial powder is equivalent to cp Ti Grade IV (ASTM F67-00). It has been produced by a hydrogenation/dehydrogenation process and supplied by SE-JONG Materials Co. Ltd. (Incheon, Korea). Fully dense titanium samples were obtained by conventional powder metallurgy technology (PM), while porous substrates were fabricated using the space-holder technique. The ammonium bicarbonate, NH4HCO3, (Cymit Química SL, Barcelona, Spain, with a minimum purity of 99.9%) with different contents (30%, 40%, 50% and 60% vol.) and two ranges of porous sizes (100–200 and 355–500 μm) were used as the spacer. These volumetric porosities and sizes of pores were selected as they are commonly used in foam Ti implants to ensure the interconnection of porosity and the in-growth of osteoblastic cells [8]. Next, the mixture of the titanium powder and the spacer was pressed to 800 MPa using a universal Instron machine to obtain green body samples (12 mm of diameter and 5 mm of height). Before sintering, the spacer was thermally removed (firstly at 60 °C and, then at 110 °C, carrying out both stages of the thermal treatment for 10 h approx. and low vacuum conditions of 10^–2^ mbar). Finally, the porous samples were sintered in a ceramic tubular furnace during 2 h at 1250 °C under high vacuum conditions (~10^–5^ mbar). The PM conditions to obtain the fully dense substrates were 1300 MPa and 1300 °C (same time and atmosphere of sintering above).

### 2.2. Electrical Impedance Spectroscopy and Porosity Characterization

Different techniques were employed to characterize the porosity at different levels (superficial and volumetric). Density measurement was performed out through the Archimedes´ method (ASTM C373-88). Total porosities (P_T_) and interconnected porosities (Pi) were determined from these measurements. On the other hand, the image analysis (IA) on the samples’ surface was carried out using a Nikon Epiphot optical microscope coupled with a Jenoptik Progres C3 camera and Image-Pro Plus 6.2 analysis software.

**Figure 1:**
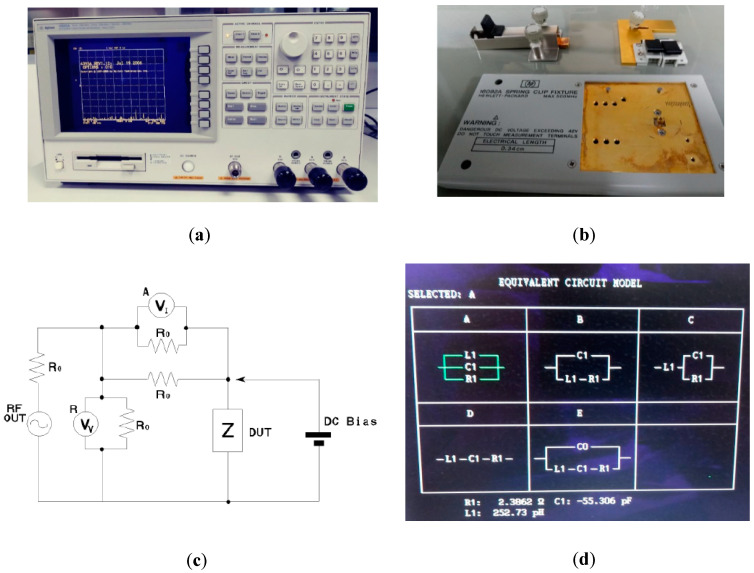
Electrical impedance analyzer and model used. (**a**) Impedance analyzer used (HP 4395A). (**b**) Clip fixture used (HP 16092A) and accessories. (**c**) Measurements’ block scheme for the impedance analyzer and Device Under Test (DUT). (**d**) Equivalent circuit model used (model A), with a resistor R_1_, a capacitor C_1_, and an inductance L_1_ in parallel, and other available options at the impedance analyzer.

The electrical impedance spectroscopy technique was used to establish the relationship between microstructural parameters (pore content and size) and the electrical properties of metal substrates. The equipment used to perform the electrical impedance measurements was the Hewlett–Packard 4395A, which is a network, spectrum, and impedance analyzer, available at IMSE-CNM-CSIC. Figure 1 shows the impedance analyzer and the different accessories used to characterize the titanium samples. The measurements’ block scheme for the impedance analyzer and Device Under Test (DUT) are also shown in Figure 1.

To place the fabricated titanium samples (device under test) on the impedance analyzer, the module HP 16092A was used, acting as a holder for the samples, in a precise and repeatable way. The impedance of each of the different titanium samples was measured at the available frequency range of the Hewlett–Packard 4395A impedance analyzer, which is from 150 kHz to 500 MHz. Measurements were carried out three times, on different days, for each sample and each frequency, to evaluate the dispersion of the measurements.

An initial electrical model has been chosen in the Hewlett–Packard 4395A impedance analyzer, to characterize the impedance measurements carried out in our titanium samples. The selected model and other available model options that were available are shown in Figure 1d. The electrical resistance R_1_ is the main parameter that the model uses to characterize changes in porosity. It is the passive element of the circuit that models the dissipation of the electric energy. A capacitor C_1_ and an inductance L_1_ are also considered, modeling two other effects, the storage of energy in electric and magnetic fields, which can also occur in the sample. The electrical resistance being the main parameter, we considered the parallel distribution of these passive elements would be a good model of the sample.

## 3. Results

### 3.1. Porous Samples Fabrication

**Figure 2:**
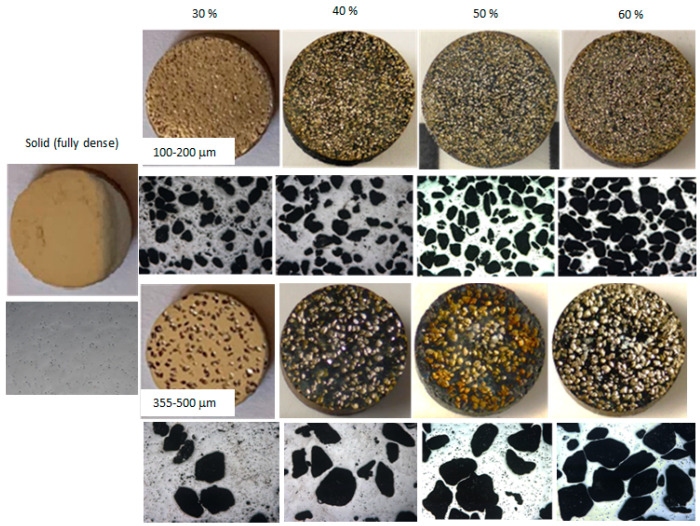
Macro- and micrographs of the titanium substrates studied. Macroscopic and microscopic appearance of the different types of titanium substrates studied (100–200 μm, upper images, and 355–500 μm, lower images), at different contents (30%, 40%, 50% and 60% vol.), in comparison with fully dense samples.

Figure 2 shows the macroscopic and microscopic appearance of the different types of titanium substrates studied, fully dense and porous samples. Furthermore, Table 1 reports the density values and more important porosity parameters obtained (P_T_, Pi, and size range of pores), using the Archimedes method and image analysis. The results indicate that there is a good correlation between the characteristics of the spacer used (volumetric porosity and range of pore size) and the porosity obtained. This fact indicates that the space-holder technique is highly recommended for manufacturing materials with a controlled porosity. The microporosity observed in the fully dense samples is typical to the conventional PM route (sintering step). However, the porous titanium substrates have two types of pores: (1) Micro pores, similar to those obtained by PM route (proportion, size, and morphology), and (2) macro pores generated by the spacer. The content, size, and morphology of the pores are responsible for the tribo-mechanical behavior (stiffness, hardness, mechanical strength, wear resistance, and scratch resistance) and corrosion resistance of porous titanium samples. On the other hand, it has been reported in the literature that pores with an adequate size allow the growth of bone tissue into the implant, as well as infiltration and adhesion of coatings [17,18], while optimal surface roughness facilitates the adhesion of osteoblasts (potential improvement of osseointegration). In this context, porous titanium parts manufactured with 50% vol. and a range of sizes between 100–200 μm have a potential better balance of biomechanical and biofunctional behavior [19].

### 3.2. Electrical Impedance Measurements

**Figure 3:**
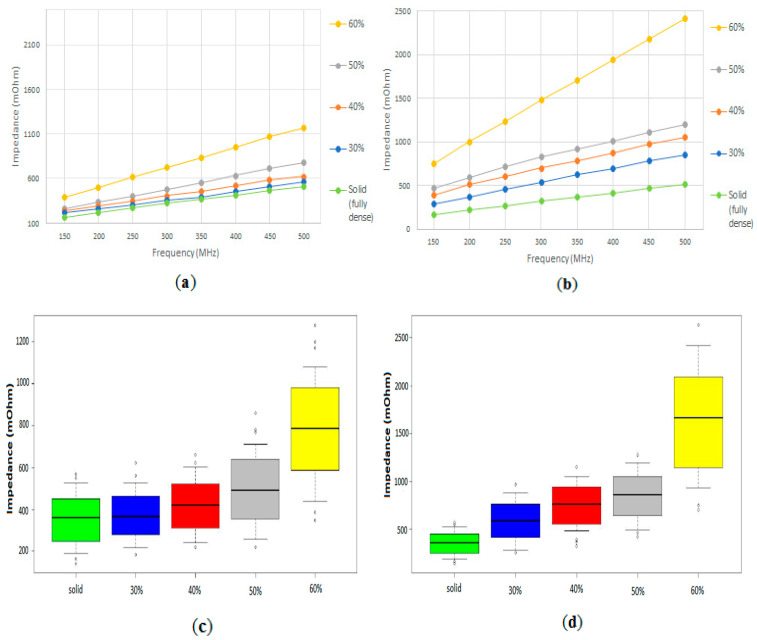
Electrical impedance measured for the porous discs. (**a**) Mean electrical impedance measured at different frequencies, for each of the 100–200 µm samples (solid, 30%, 40%, 50% and 60% volume porosity). (**b**) Mean electrical impedance measured at different frequencies, for each of the 355–500 µm samples. (**c**) Box and whiskers plot for each of the measurements of the 100–200 µm samples, at all frequencies, showing the median, lower, and upper quartiles, and highest and lowest observations. (**d**) Box and whiskers plot for each of the measurements of the 355–500 µm samples.

Figure 3 shows the electrical impedance measured for the porous discs of 100–200 μm and for the porous discs of 355–500 μm, at different frequencies (from 150 to 500 MHz). As can be seen, in both cases the electrical impedance of the samples increases with increasing frequencies, and there are significant differences between the samples with different porosity volumes and the fully dense samples. In Figure 3c,d, box and whiskers plots for each of the measurements of both groups of samples, at all frequencies, are shown, indicating the median, lower, and upper quartiles, and highest and lowest observations.

We can observe that there is a higher dispersion of the measurements for the samples with higher porosities, especially for the 60% volume samples. Of the different frequencies used, 500 MHz would be the optimal to characterize and differentiate the level of porosity and the size of pore used. Samples with higher size of pores (355–500 μm) were more sensitive to the variation of impedance. The technique used could be an appropriate technique to characterize the level of porosity of the titanium samples and size of pore, with the different volumetric porosity percentages (30%, 40%, 50% and 60%) and ranges of pore size (100–200 and 355–500 mm) used.

## 4. Discussion

### 4.1. Characterization of Volumetric Porosity and Pore Size

In accordance with the experiments reported in [10], higher pore size resulted in higher impedance. An increasing pore size limited electron mobility and therefore increased the electrical impedance of the sample. However, on the contrary to the experiments reported in [10], where the pore size had a minor influence on the electrical conductivity of porous titanium and Ti6A14V, our experiments showed an important difference between the sizes of 100–200 and 355–500 μm. As can be seen in Figure 1, the impedance measured for 355–500 μm is approximately double the impedance measured for 100–200 μm, for most frequencies and volumetric porosities.

**Figure 4:**
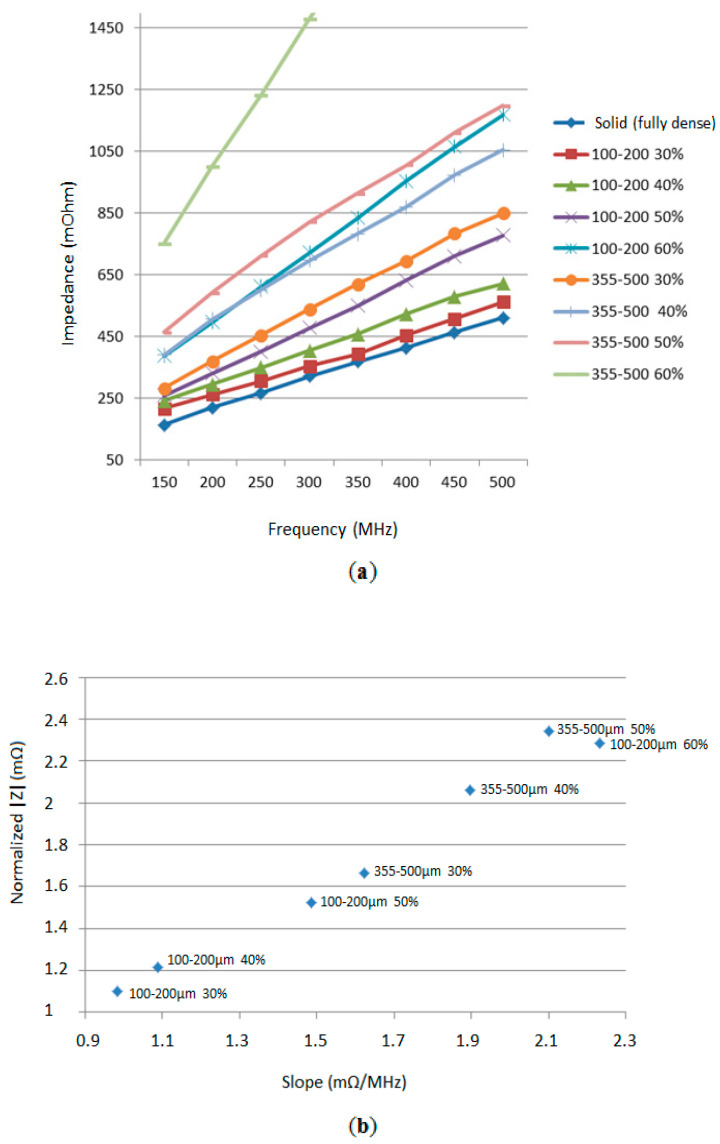
Discrimination of specimens. (**a**) Mean electrical impedance measured at different frequencies, for all samples and frequencies. (**b**) Normalized impedance (mΩ) versus measured slope (mΩ/MHz) in the range of measured frequencies (150 to 500 MHz).

Furthermore, the effect of frequency on the measured electrical impedance was measured. As previously commented, the electrical impedance of the samples increased with increasing frequencies, and there was a higher difference of measurements for different volume samples at higher frequencies, which made these frequencies (around 500 MHz) optimal to characterize and differentiate the level of porosity and the size of pore used. Figure 4a shows the mean electrical impedance values measured for all samples at all frequencies explored. We can observe that different samples can be univocally discriminated from each other with the impedance spectroscopy analysis carried out (sweep in frequencies from 150 to 500 MHz), with the observation of the slope (mΩ/MHz) and absolute magnitude (mΩ) of the obtained impedance spectrometry. Figure 4b shows the normalized impedance (division of the impedance values obtained by the solid sample impedance) versus the slope obtained for the different samples (mΩ/MHz), showing the potential implementation of a classifier based on these parameters for the characterization of the samples.

It is also important to discuss and compare the results of our technique with other established techniques in the field of material characterization and porous titanium works. In our work we used two of the most established techniques used to characterize the porosity at different levels (superficial and volumetric porosities): The Archimedes´ method (ASTM C373-88), and image analysis (IA) on the samples surface, as described in the Materials and Methods section. As we can see in Table 1, and in accordance with other previous works [20,21,22,23], there are many cases where neither of the two methods is effective to discriminate the volumetric porosity or the pore size range, lacking statistical significance. Our results, and this more detailed comparison with the existing techniques, showed the efficiency and advantages of the proposed method in the characterization of porous titanium samples, in a precise, simple, and affordable way.

On another hand, the equivalent impedance, Z_eq_, according to the model selected (Figure 1d) is given by Equations (1) and (2):

(1) $$\frac{1}{Z_{\mathrm{eq}}}=\frac{1}{R_{1}}+\frac{1}{{\mathrm{jwL}}_{1}}+\frac{1}{\left(-\frac{j}{{\mathrm{wC}}_{1}}\right)}$$

(2) $$Z_{\mathrm{eq}}=\frac{R_{1}{\mathrm{wL}}_{1}}{{\mathrm{wL}}_{1}+j\left(R_{1}C_{1}L_{1}w^{2}-R_{1}\right)}$$

**Figure 5:**
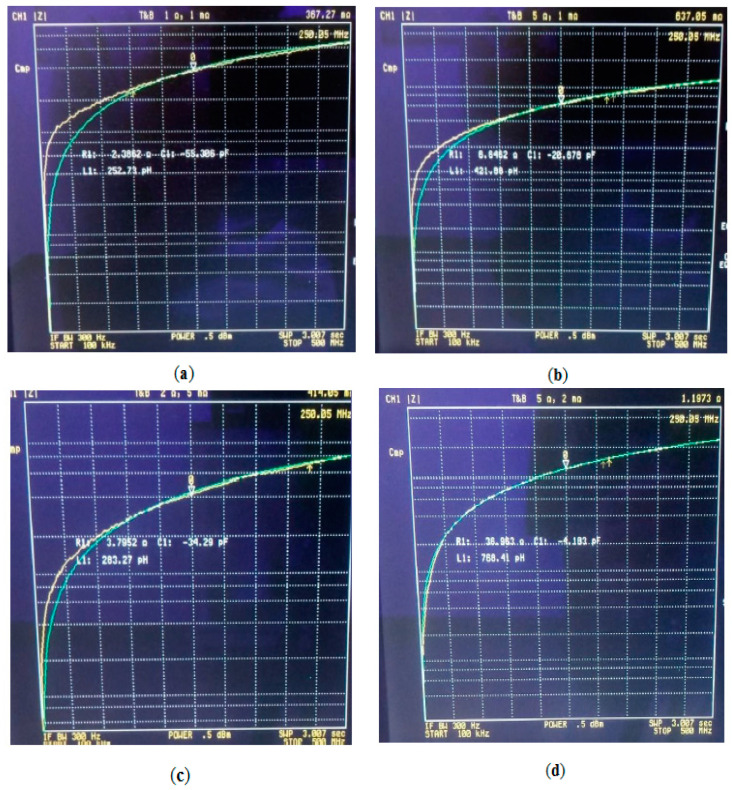
Experimental data versus theoretical model results (modulus of impedance vs. frequency). The yellow line shows the experimental values obtained, and the green line represents the values obtained by the theoretical equivalent circuit selected, in the range of frequencies explored. (**a**) Size of pore of 100–200 µm and 30% of porosity volume. (**b**) Size of pore of 100–200 µm and 60% of porosity volume. (**c**) Size of pore of 355–500 µm and 30% of porosity volume. (**d**) Size of pore of 355–500 µm and 60% of porosity volume. There is a very good correlation in this case between theoretical values and experimental values for all frequencies. Mean values obtained for R_1_, L_1_, and C_1_ in the model are summarized in Table 1.

The mean experimental values obtained for R_1_, L_1_, and C_1_ for the selected model, in each of the samples, are shown in Table 2. In addition, as shown in Figure 5, there is a good match between the experimental values and the theoretical values calculated by the Hewlett–Packard 4395A impedance analyzer, with the different values for R_1_, L_1_, and C_1_, at the different frequencies. As can be seen in Figure 5, there is a good match in all cases, especially at higher frequencies. For the case of size of pore of 355–500 µm and 60% of porosity volume, the theoretical model fits very well the experimental values for all frequencies.

We can observe again the statistical significance of the different values obtained, which enabled us to univocally characterize the different specimens in a precise way, in opposition with the discrimination that can be found with the Archimedes´ method or image analysis (Table 1).

### 4.2. Real Time Monitoring of Biological Osseointegration and Potential Use in Medical Sensors

Different studies on titanium implants have shown that at least a 100 μm average pore size is necessary to mark and guide the cellular response to produce and synthetize bone matrix [8,24,25,26]. Porous titanium parts manufactured with 50% vol. and a range of sizes between 100–200 μm have a potentially better balance of biomechanical and biofunctional behavior [19]. This bone in-growth through the pores also promotes long term stability and implant osseointegration [8].

**Figure 6:**
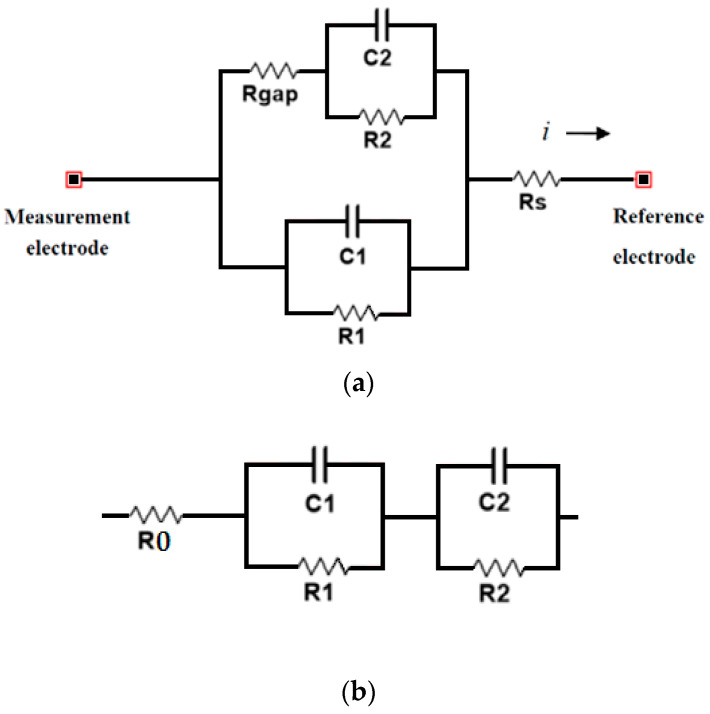
Electrical circuits used to model interfaces between impedance measurement electrodes and biological structures. (**a**) Electrical circuit used in [28], where Rgap models the resistance between the cell layer and the electrodes, Rs is the resistance between the cell layer and the reference electrode, R_1_ and C_1_ form the impedance of the area of the electrodes that is not covered by cells, and R_2_ and C_2_ model the impedance of the area of the electrodes covered by cells. (**b**) Electrical circuit used in [3], where R_0_ models the overall electrolyte bulk behavior, R_1_ and C_1_ model the slower electrochemical phenomena (such as polarization due to mass diffusion), and R_2_ and C_2_ model the faster electrochemical phenomena (such as the polarization due to charge transfer).

The use of electrical impedance spectroscopy has been studied for cell culture monitoring in different works for a wide variety of applications, such as the study of cell proliferation [1,27,28,29], cell toxicity [30], or cellular differentiation [31]. In [28], an oscillation-based circuit was proposed for the measurement of electrical impedance in cell cultures. Figure 6a shows the equivalent model proposed in [28] for modeling the electrical behavior of the cell culture and instrumentation system.

On another hand, in [3], electrical impedance spectroscopy was used for the characterization of the interface between bone and prosthesis. In vitro experimental results pointed out positive indications for the full method implementation by highlighting its capability to detect the presence of a satisfying connective tissue, as well as the diagnosis of its thickness and its nature. Figure 6b shows the equivalent electric circuit used to model electrochemical phenomena between the bone and the prosthesis. In [16], the work was continued with the design of a microcontroller-based prototype conceived as a stand-alone instrument to be used for in vivo clinical applications. The preliminary laboratory tests showed positive results, although there was calibration in the operating range, as well as adaptation to in vivo situations were still necessary.

The implementation of a medical sensor that could monitor the state of the implant in an affordable and real time way needs to integrate these different approaches, in order to efficiently characterize porosity level, cell culture growth and osseointegration, and possible corrosion processes or fractures that can occur inside the implant. An integrated circuit approach could be followed, making use of the different electrical models for the decoding of the measured impedance values. The medical sensor could also be improved with the design and implementation of microelectrode arrays (MEAs) that could characterize the 3D spatial distribution of the implant porosity with impedance spectroscopy, in a similar way as has been explored in other works for other applications [5,32], to perfectly verify the manufacturing process of the implant in 3D, and to address possible practical issues to measure the properties of the titanium materials after they have been implanted in patients. Furthermore, it would also be interesting to verify if impedance spectroscopy could also be used to characterize corrosion processes or fractures that could occur inside the implant, which have been studied by other methods [12,20], or to characterize the process of adhesion of osteoblasts and bacteria on implants [33].

## 5. Conclusions

In this work, we explore the use of impedance spectroscopy for the characterization of porous titanium samples, as a potential technique for the real-time measurement of bone implants. Initial experiments have been carried out with the Hewlett–Packard 4395A impedance analyzer on different titanium samples, of different volumes of porosities and different sizes of pores.

The electrical impedance of the samples increases with increasing frequencies. Significant differences between the samples with different porosity volumes and the solid samples were found, being samples with a higher size of pores (355–500 μm) more sensitive to the variation of impedance. Higher frequencies (around 500 MHz) also showed a better sensitivity to impedance changes. An electric model was proposed to model the experimental results.

Different samples could be univocally discriminated from each other with the impedance spectroscopy analysis carried out (sweep in frequencies from 150 to 500 MHz), with the observation of the slope (mΩ/MHz) and absolute magnitude (mΩ) of the obtained impedance spectrometry. Impedance spectroscopy therefore proved to be an appropriate technique to characterize the level of porosity of the titanium samples and pore size, for different practical applications such as implant assessment, in a quick, affordable, and non-invasive way.

Smart implants would require affordable sensors that could monitor in real time the state of the implant, possible structural problems, and integration with biological structures. Further work is needed to design and implement microelectrode arrays (MEAs) that can characterize the 3D spatial distribution of the implant porosity with impedance spectroscopy, to perfectly verify the manufacturing process of the implant, and to address possible practical issues to measure the properties of the titanium materials after they have been implanted in patients. Furthermore, it will also be interesting to verify if impedance spectroscopy can also be used to characterize corrosion processes or fractures that can occur inside the implant, or to characterize the process of adhesion of osteoblasts and bacteria on implants.

## Figures and Tables

**Table 1:** Density and porosity features obtained by the Archimedes method and image analysis.

Spacer		Archimedes Method			Image Analysis	
		Density (g/cm^3^)	Porosity (%)		P_T_ (%)	Size Range (µm)
Volumetric Porosity (%)	Size Range (µm)		Total	Interconnected		
30	100–200	3.14 ± 0.02	30.2 ± 0.2	18.0 ± 0.1	31.2 ± 0.6	192 ± 117
	355–500	3.15 ± 0.01	30.0 ± 0.19	19.0 ± 0.1	24.6 ± 2.5	435 ± 401
40	100–200	2.69 ± 0.02	40.2 ± 0.6	32.9 ± 0.8	42.1 ± 3.3	226 ± 178
	355–500	2.67 ± 0.02	40.8 ± 0.5	27.9 ± 0.7	43.7 ± 7.8	359 ± 223
50	100–200	2.37 ± 0.01	47.4 ± 0.1	46.7 ± 0.1	58.4 ± 4.2	217 ± 154
	355–500	2.14 ± 0.01	52.5 ± 0.3	50.8 ± 0.3	56.6 ± 6.9	278 ± 322
60	100–200	1.97 ± 0.02	56.4 ± 0.5	51.8 ± 1.3	61.0 ± 2.4	295 ± 287
	355–500	1.90 ± 0.04	57.8 ± 0.5	53.0 ± 0.9	55.5 ± 10.3	302 ± 332

**Table 2:** Mean values obtained for R_1_, L_1_, and C_1_ in the selected model, for each of the samples.

	Range of Pore						
Porosity volume		**100–200 µm**			**355–500 µm**		
		R_1_(mΩ)	L_1_(pH)	C_1_(pF)	R_1_(mΩ)	L_1_(pH)	C_1_(pF)
	30%	2386.2 ± 0.1	252.73 ± 0.01	55.31 ± 0.01	3795.2 ± 0.1	283.27 ± 0.01	34.29 ± 0.01
	40%	3853.2 ± 0.1	307.34 ± 0.01	36.29 ± 0.01	14578.6 ± 0.1	443.12 ± 0.01	26.34 ± 0.01
	50%	5298.1 ± 0.1	375.29 ± 0.01	27.27 ± 0.01	27817 ± 0.1	603.70 ± 0.01	17.39 ± 0.01
	60%	6646.2 ± 0.1	421.88 ± 0.01	20.68 ± 0.01	36963 ± 0.1	768.41 ± 0.01	4.18 ± 0.01
	Fully dense	R_1_ = 1095.5 ± 0.01 mΩ L_1_ = 169.88 ± 0.01 pH C_1_ = 53.528 ± 0.01 pF					
